# Temporal Analysis of Image-Rivalry Suppression

**DOI:** 10.1371/journal.pone.0045407

**Published:** 2012-09-25

**Authors:** Rishi Bhardwaj, Robert P. O’Shea

**Affiliations:** 1 Department of Psychology, University of Otago, Dunedin, New Zealand; 2 Department of Psychology, Durham University, Durham, United Kingdom; 3 Nethradhama School of Optometry, Bangalore, India; 4 Discipline of Psychology and Cognitive Neuroscience Research Cluster, School of Health and Human Sciences, Southern Cross University, Coffs Harbour, Australia; Nothwestern University, United States of America

## Abstract

During binocular rivalry, perception alternates between two different images presented one to each eye. At any moment, one image is visible, *dominant*, while the other is invisible, *suppressed.* Alternations in perception during rivalry could involve competition between eyes, *eye-rivalry*, or between images, *image-rivalry*, or both. We measured response criteria, sensitivities, and thresholds to brief contrast increments to one of the rival stimuli in *conventional rivalry* displays and in a display in which the rival stimuli swapped between the eyes every 333 ms–*swap rivalry*–that necessarily involves image rivalry. We compared the sensitivity and threshold measures in dominance and suppression to assess the strength of suppression. We found that response criteria are essentially the same during dominance and suppression for the two sorts of rivalry. Critically, we found that swap-rivalry suppression is weak after a swap and strengthens throughout the swap interval. We propose that image rivalry is responsible for weak initial suppression immediately after a swap and that eye rivalry is responsible for the stronger suppression that comes later.

## Introduction

To understand our conscious experience of the visual world, researchers have increasingly turned to rivalry phenomena [1]. In these, conscious experience changes irregularly every few seconds or so among two or more possibilities despite there being no corresponding change in what is presented to the eyes [2]–[7], thereby making them candidates for finding the neural correlates of consciousness [8]. In this paper, we focus on binocular rivalry in which two incompatible stimuli are imaged on corresponding retinal regions of the two eyes. One stimulus is visible for few seconds (*dominant)*, while the other stimulus is invisible (*suppressed)*; after a few seconds, visibility reverses, often with a brief, intervening, unstable composite of the two stimuli [for reviews see 9–10].

The neural processes mediating rivalry are thought to occur at multiple cortical areas such that competition takes place between the representations of the two images tagged with low-level, eye-of-origin information–*eye rivalry,* [11]–[17] and between representations of the two images at some higher level of the visual system–*image rivalry* [3], [18], [19]. Both forms of rivalry presumably occur during conventional binocular rivalry, in which one image is shown continuously to one eye and the other image is shown continuously to the other. Image rivalry must be involved during *swap rivalry*, in which the images swap between the eyes about every 250 to 500 ms while observers report irregular, much-longer alternations between the two images [3], [20].

One technique to understand what happens to a suppressed stimulus during binocular rivalry is to measure the strength of suppression by delivering a probe to one of the rival stimuli during its dominance and suppression phases [14], [21]–[28]. The ideal probe is a variation in some aspect of one of the rival stimuli, such as contrast, because this ensures that the probe is processed by the same neurons that are processing the rival stimulus [25], [27]. The difference in the threshold to detect the probe stimulus during dominance and suppression gives an estimate of strength of suppression.

Recently Bhardwaj, O’Shea, Alais, and Parker [28] measured strength of suppression during both conventional rivalry and swap rivalry to contrast increments of one of the rival stimuli. They found strong suppression during conventional rivalry and weak suppression during swap rivalry. They concluded that conventional rivalry involves both eye rivalry and image rivalry and that swap rivalry involves mainly image rivalry.

In this paper, we set out to make three tests of the conclusion of Bhardwaj et al. [28] that image-rivalry suppression is weak:

In Experiment 1, we set out to replicate the finding of Bhardwaj et al. in case it represented a Type-I statitical error. We were able to replicate the finding.

In Experiment 2, we tested the possibility is that the findings of Bhardwaj et al. were contaminated by differences in response criteria between swap rivalry and conventional rivalry (although we concede that Bhardwaj et al. reproduced their finding of weak suppression with a forced-choice procedure). We used the Theory of Signal Detection (TSD) [29] to measure response criteria and perceptual sensitivities to probes of various contrasts.

We were motivated to assess response criteria by Caetta, Gorea, and Bonneh’s research [30] into motion-induced blindness, a perceptually bistable phenomenon in which peripheral dots superimposed in a globally moving background disappear and reappear [31]. They found observes to have a stricter response criterion during suppression than during dominance. The virtue of TSD is that perceptual sensitivities are independent of response criteria. Moreover, although sensitivity during conventional binocular rivalry has been measured in the past [26], [32] none of the previous studies have reported response criteria.

We found no differences in response criteria for the dominance and suppressed phase of either conventional or swap rivalry, reassuring us about the conclusion of Bhardwaj et al. [28]. Overall response criteria were more conservative during conventional rivalry than during swap rivalry.

In Experiment 2, we also tested the conclusions of Bhardwaj et al. [28] by presenting probes with a short, fixed delay after an observer’s key press, that is, with a relatively short and fixed delay from the onset of perceptual dominance. This is unlike the procedure of Bhardwaj et al., who waited for various delays after the onset of perceptual dominance so that probes were always presented at the onset of a swap of one image to the left eye. Such delays could have meant that perception reversed before the probe could be delivered, artifactually weakening suppression. This would be more likely for swap rivalry than for conventional rivalry because the former’s durations of suppression are shorter [3], [20].

We found that strength of suppression was similar from swap rivalry and from conventional rivalry. Although this similarity could be taken to refute the conclusions of Bhardwaj et al. [28] a post-hoc analysis suggested that suppression in swap rivalry was weak immediately after the swap as found by Bhardwaj et al., but strengthened about 150 ms later. This temporal dependence made the data noisy because probes were presented at various delays relative to the swap.

In Experiment 3, we thus manipulated the delay between the swap and the presentation of the probe. We introduced the probe immediately after the swap, after 100 ms, or after 200 ms (probes reached full contrast 57 ms after being introduced). We refer to these times as early, middle, and late in the interval between one swap of the stimuli and the next (our swap interval was 333 ms). We found weak suppression early in the swap interval, similar to that found by Bhardwaj et al. [28] and in Experiment 2, and strong suppression later, similar to that of conventional rivalry.

We propose that immediately after a swap, suppression is mainly accomplished by image rivalry, and that later in the swap interval it is augmented by eye rivalry.

## Experiment 1

In Experiment 1, we set out to replicate the findings of Bhardwaj et al. [28].

### Materials and Methods

The research in all Experiments was approved by the Ethics committee of the University of Otago. All participants gave their consent to take part in the study by signing the required informed consent form.

#### Observers

Three observers (two males, MJB, and RB [one of us], and one female, LC) participated. All observers had corrected-to-normal visual acuity and normal stereoacuity. Ages ranged from 20 to 27 years. Observer MJB had 4–6 prism diopters of exophoria for near, although he had no trouble maintaining binocular alignment of the rival stimuli. MJB and LC were naïve, inexperienced psychophysical observers who were paid for their participation.

#### Apparatus

Stimuli were generated by an Apple Power Macintosh G-4800 using Matlab in conjunction with the Psychophysics Toolbox [33], [34]. Stimuli were displayed on a Sony Trinitron high-resolution, 19-inch, color monitor (CPD-Experiment 230) at a viewing distance of 57 cm. The monitor’s frame rate was 75 Hz and screen resolution was 1024×768 pixels. Its screen was calibrated and linearized using a Minolta Chroma meter (model CS-100). Stimuli for the left eye were presented on the left half of the monitor screen and stimuli for the right eye on the right half. The observer used a mirror stereoscope to bring the two views into alignment. Observers responded using the computer keyboard.

#### Stimuli

**Figure 1:**
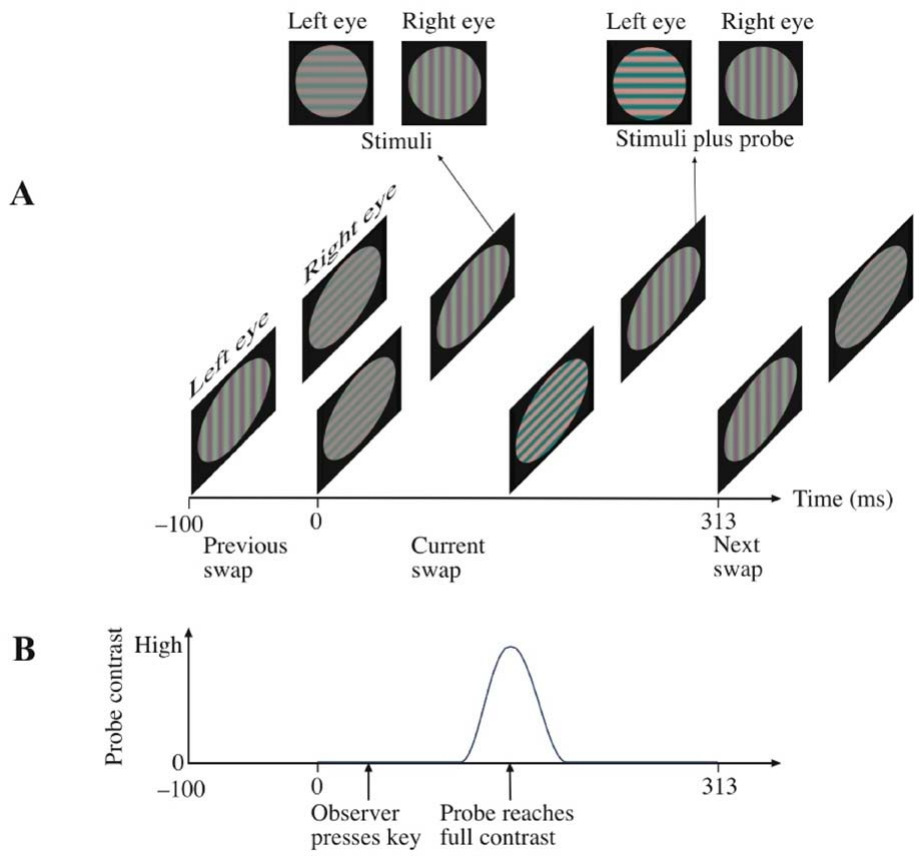
Illustration of stimuli and procedure during flicker-and-swap (FS) rivalry for a trial when a probe was delivered (panel A). For simplicity, we have shown neither that the stimuli were flickering on and off at 18 Hz nor that the screen went black 100 ms after the presentation of the probe until the observer had responded. Illustration of the time course of the probe (panel B).

The rival stimuli were circular patches of 7 cycles per degree of visual angle sinusoidal grating with a diameter of 2.34 degrees (illustrated in Figure 1A). One was a red horizontal grating (CIE chromaticity coordinates x = 0.315; y = 0.321); the other was a green vertical grating (x = 0.270; y = 0.347). Both gratings had 25% contrast and a mean luminance of 13 cd/m^2^. To assist proper alignment of both eyes, there were two vertical bars on either side of the stimuli. These subtended 2.62 deg high and 0.13 deg wide. An inner pair of gray bars had a luminance of 0.16 cd/m^2^ and was placed with their centres 1.96 degrees from the centre of the grating. An outer pair of white bars had a luminance of 0.30 cd/m^2^ and was placed with their centres 2.13 degrees from the centre of the grating. All these stimuli were displayed on an otherwise uncontoured background with a luminance of 0.05 cd/m^2^. The experiment was performed in a dark room; the only significant source of light came from the monitor screen.

The rival stimuli could be presented in three different ways:

1. Static unchanging, conventional rivalry grating stimuli (Static).
2. Flicker-only (FO). These were similar to static stimuli but flickered on and off at 18 Hz.
3. Flicker-and-swap (FS). These were similar to FO but swapped between the eyes at 1.5 Hz (as shown in Figure 1A).

Static and FO allow eye rivalry, whereas FS supposedly allows only image rivalry.

The probe was a superimposed red grating with the same spatial frequency, phase, size, and orientation as the red test grating, yielding a contrast increment to that rival grating. To avoid abrupt onset and offset of the probe, the probe was ramped on and off using a Gaussian temporal contrast envelope with a half-height full-width of 75 ms (see Figure 1B).

### Procedure

We used the stimuli and procedure of Bhardwaj et al.’s [28] Experiment 3 to measure the contrast-increment threshold during dominance and suppression for each test condition for every observer.

Observers controlled the onset of the probe. We asked them to trigger the probe stimulus either when the red horizontal grating was completely dominant or when the green vertical grating was completely dominant. If, after deciding to press the key to signal dominance of a particular rival stimulus, the observer’s perception alternated to the other rival stimulus before the key could be pressed, we asked the observers to abort the trial by pressing another key.

Observers responded to the probe by pressing a second key if they saw it and a third key if they did not. After observers had given their responses, the computer provided feedback for a correct response with a single short tone and for an incorrect response with two short tones. We also gave general feedback after each session. We gave feedback to help observers achieve and maintain optimal performance and to replicate the procedure of Bhardwaj et al. [28].

In FS conditions, when the observer depressed a key, the program waited until the start of the next swap of the red grating to the left eye to show the probe. This meant that the onset of the probe was subject to a random delay between 13 ms (1 frame) and 666 ms (50 frames) after the key press. There were similar random delays in the Static and FO conditions. In all conditions, the contrast of the probe rose to its full value then fell back to its original value with a Gaussian profile, the whole contrast pulse taking 107 ms. After the probe finished, the original stimuli were re-presented for 107 ms, then the screen went black for the observer to respond.

We varied the contrast of the probe with an adaptive QUEST procedure [35] to find the 75% threshold level. On 50% of the trials the probe was presented and on the remaining 50% of the trials the probe was not presented. Two interleaved staircases comprising 20 trials each were used. These staircases were preceded by four practice trials. Observers completed five sessions. A single session consisted of the three rivalry conditions, each with blocks of dominance and suppression. The order of the test conditions within a session was randomized and the order of testing dominance and suppression was random for each observer then alternated over sessions.

### Results and Discussion

We give the mean thresholds for dominance and suppression in Figure S1. We then computed the strength of suppression using Equation 1:
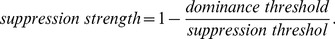
(1)


**Figure 2:**
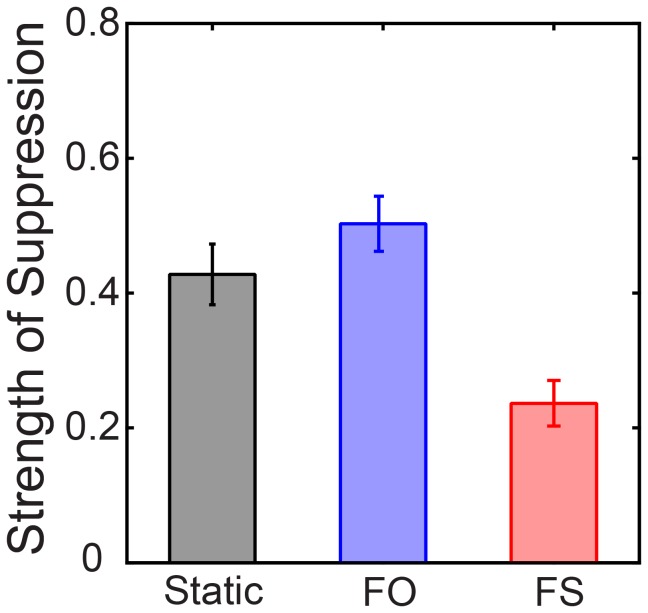
Mean strengths of suppression for different rivalry conditions from Experiment 1. Error bars show 1 standard error of the mean.

Suppression strengths can range from 0 (no suppression) to 1 (complete suppression). Equation 1 is the same as used by Bhardwaj et al. [28]. We found strengths of suppression were similar to those found by Bhardwaj et al [28]: Strong suppression for static and FO rivalry (0.43 and 0.50 respectively) and weak suppression for FS rivalry (0.24). These differences were significant, *F*(2, 4) = 10.46, *p<*.05 and are shown in Figure 2.

The results shown in Figure 2 are essentially identical to those of Bhardwaj et al. [28]. We conclude that those results are reliable.

## Experiment 2

### We had Two Aims in Experiment 2

1. To measure response criteria during both conventional and swap rivalry.
2. To measure strength of swap-rivalry suppression, based on sensitivity, with probes having a fixed, short delay after the observer’s key press.

### Materials and Methods

The observers of Experiment 2 were the same as in Experiment 1, except that there were two other naïve, females: AP and FR who took part only at a contrast increment level of 0.27 (see later). The apparatus, and stimuli of Experiment 2 were identical to those of Experiment 1 but the procedure differed in the following ways: For the three observers from Experiment 1, we used the average of each observer’s dominance and suppression thresholds for each condition from Experiment 1 to give one value of contrast to be tested for each condition and observer. We also tested at three values above and below this average value in 0.04 log steps of contrast, giving seven contrast levels for each condition and observer.

When the observer depressed a key, there was a fixed delay of 57 ms before the contrast increment began. In FS rivalry the probe was presented either to the left eye or to the right eye, depending on to which eye the to-be-probed stimulus was being shown. If there was not enough time to show the probe to a single eye during FS conditions then the probe swapped between the eyes at the same time as the gratings swapped. To equate the probe presentation to both eyes during FO and static rivalry, the rival stimuli were randomly interchanged between the eyes during the inter-trial interval on half of the trials. Observers were not aware that the stimuli were interchanged between the two eyes during FO and static conditions.

A single session consisted of all the three conditions within each of which were blocks of dominance and suppression. The order of the conditions within a session was randomized and the order of blocks was random for each observer then alternated over sessions. There were 55 trials during a single run; the first five were practice trials in which the probe was always presented. Of the 50 remaining trials, randomly on half of the trials the probe was presented and on the remainder the probe was not presented.

The order of testing the seven contrast values was randomized without replacement over seven sessions. These sets of seven sessions were repeated until all observers completed 10 sets. Prior to commencing the first set, all observers also completed at least four practice sessions at each contrast level.

Observers responded by pressing one key if they saw the probe and another key if they did not. We used a Yes/No procedure because we wanted to keep the response procedure similar to that used by Caetta et al. [30] to allow us to compare results from rivalry and from motion-induced blindness.

Observers AP and FR had a similar procedure, except that their probes had only one contrast of 0.27. We ran these observers to increase power of statistical analyses of this contrast value, which was common for all observers.

### Results and Discussion

We computed two measures for each observer at each contrast level, perceptual state, and stimulus condition. One was the TSD measure of *response criterion* using Equation 2 (from Macmillan and Creelman’s [36] formula):
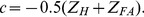
(2)where *Z_H_* and *Z_FA_* are the *z* scores of hits and false alarms. A negative value denotes a liberal response criterion (a greater willingness to say that the probe had been presented), and a positive value denotes a conservative response criterion (a greater willingness to say that the probe had not been presented).

The other was *sensitivity* using Equation 3 (from Macmillan and Creelman’s [36] formula):
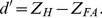
(3)


Sensitivities range from 0 (chance responding) to infinity (perfect accuracy). From the sensitivities, we also calculated strength of suppression with Equation 1.1:



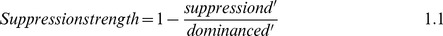



These suppression strengths are similar to those yielded by Equation 1.

Because each observer responded to his or her own contrast levels, to combine data across observers for statistical analyses, we coded contrast increment as numerical values ranging from 1 to 7. A contrast value of 1 means the minimum contrast for each condition for each observer, a contrast value of 4 means the threshold contrast for each condition for each observer, and a value of 7 means the maximum contrast for each condition for each observer. This allowed us to have a factor of contrast with seven levels, along with the other factors of perceptual state and type of stimulus, for an analysis of variance (ANOVA).

#### 

##### Response criterion

We pooled the data from the 10 sessions to calculate the response criteria for each observer in all the conditions. We analyzed the data with a three-way, within-subject ANOVA with condition, perceptual state, and contrast as factors. The only significant effect was that of condition, F(2, 4) = 8.53, p<.05. This arises because observers had more conservative criteria (higher values of c) during static, less conservative criteria during flicker-only, and least conservative for flicker-and-swap (see Figure S2, also see Figure S3’s top panel for individual observer data). Overall, response criteria were significantly greater than zero (zero is a neutral response criterion) for static rivalry and for flicker-only rivalry, t(41) = 6.28 and 2.74, ps <.01, but not for flicker-and-swap rivalry, t(41) = –0.43, p>.05.

**Figure 3:**
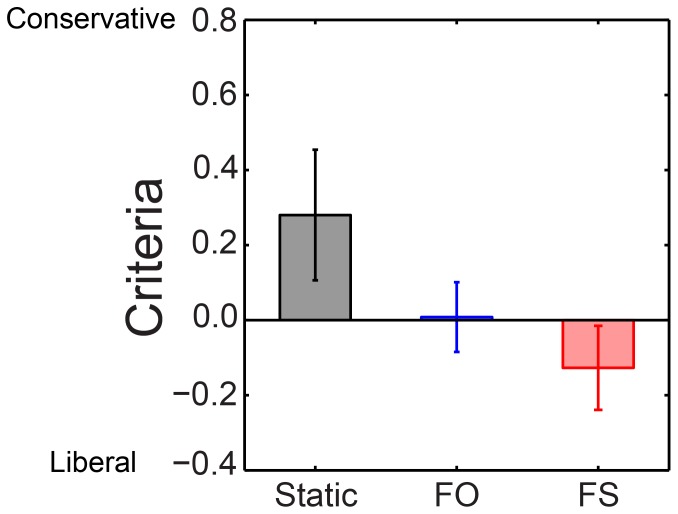
Response criteria measure for different rivalry condition in Experiment 2 at the common contrast increment of 0.27 (n = 5) for all test conditions. Error bars show ±1 standard error of the mean. The differences are significant: Criteria are conservative (observers are unlikely to say the probe was presented) for Static rivalry, neutral for FO, and liberal for FS.

To confer greater statistical power, we analyzed response criteria at the common contrast value of 0.27 with a three-way within-subject ANOVA of five observers (see Figure S3’s bottom panel for individual observer data). Again the only significant effect is that of condition, *F*(2, 4) = 7.05, *p*<.05. This difference, shown in Figure 3 confirms that observers have more conservative criteria during static, less conservative criteria during flicker-only, and least conservative criteria for flicker-and-swap conditions.

Unlike Caetta et al. [30] we did not find any consistent criterion difference between dominance and suppression for any of the conditions. This probably happened because we gave feedback after every trial, whereas Caetta et al. [30] did not. (We provided feedback after every trial because that is what Bhardwaj et al. [28] did.) Trial-by-trial feedback would tend to minimize criterion differences. Critically, it means we cannot attribute differences in sensitivity during dominance and suppression to different response criteria.

The overall differences in criteria among the three conditions could be due to task difficulty. Because we gave feedback on every trial, observers would have known when they responded “no” erroneously. When conditions were difficult (e.g., when there was 18 Hz flicker and 1.5 Hz swapping) observers almost never saw the probe. Feedback may have prompted them to look for any excuse to say “yes”–to adopt a liberal criterion. Yet when conditions were easy (i.e., when there was no 18 Hz flicker and no 1.5 Hz swapping), there would have been no such pressure, because observers could see the probe frequently and were able to say “yes” frequently, allowing them to adopt a conservative response criterion [cf. 37].

##### Strength of suppression

We pooled the data from the 10 sessions to calculate the d′ for each observer in all the conditions so that each value for each observer was based on 500 trials. We give details of these analyses in the Supporting Information (see Item S1 for details of the analysis, Figure S4 for individual data, and Figure S5 for mean data). From these we calculated strength of suppression (Equation 1.1).

**Figure 4:**
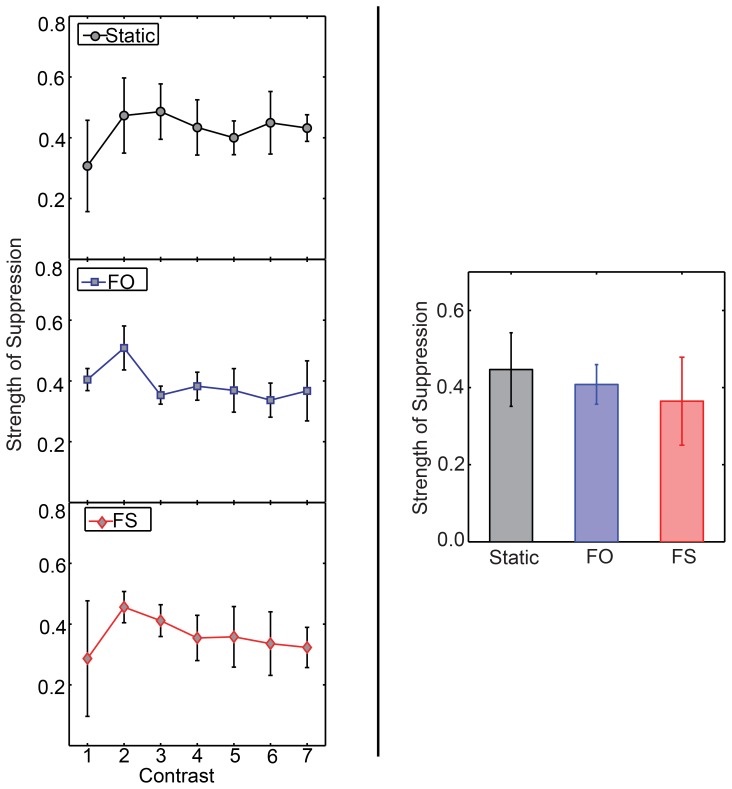
Strength of suppression for different rivalry conditions in Experiment 2. The panels on the left show the strength of suppression for static (top), FO (middle), and FS (bottom) as a function of contrast (*n* = 3). The panel on the right shows the strength of suppression for these conditions for a contrast increment of 0.27 (*n* = 5). Error bars show 1 standard error of the mean. There are neither significant differences among contrasts nor among the three rivalry conditions.

We analysed strength of suppression using a two-way within-subject ANOVA with condition and contrast as factors. We have plotted the means in the left panel of Figure 4 (see Figure S7 for individual observer data). We did not find any significant main effects or interaction. When we analysed all participants’ data at common contrast increment of 0.27 with a one-way within-subject ANOVA we also found no significant effects, as shown in the right panel of Figure 4 (see Item S2 for details of the analysis and Figure S6’s bottom panel for individual observer data). What can be seen from this graph is that the strength of suppression in each condition is around 0.4. Although the means go in the same direction as found by Bhardwaj et al. [28], the differences are not significant, possibly because of high variability in the FS condition.

One interpretation of this result is that there is no difference in the strength of suppression among any of the conditions and that the differences found by Bhardwaj et al. [28] are an artefact of delayed probes’ being delivered during the wrong perceptual state during FS rivalry. However, although this could make some contribution to the lack of difference among the conditions, we doubt it is the only contribution for two reasons:

1. We calculated that there were not enough probes delivered to the wrong state to account for all of the weakening of suppression in swap rivalry. To do so, we first estimated the mean duration of dominance/suppression in FS conditions using the results of Logothetis et al. [3]: 2170 ms. (We did this because we did not have mean duration of dominance/suppression from any of our Experiments.) If so, such a mean duration is much longer than the mean probe delay of 333 ms. When we fitted the distribution of these episodes of dominance/suppression to a gamma function, as done by Logothetis et al., we found that only 1% of episodes would have involved a probe that was delivered during the wrong state. Yet to halve the strength of suppression in FS, assuming it is really the same as that from conventional rivalry, from probes being delivered to the wrong state, 19% of probes would have had to be so delivered (see the Item S4 for how we derived this value).
2. Other research, using a similar procedure of delivering probes with a short, fixed delay after a participant’s key press, also found weaker suppression in FS conditions than in conventional rivalry [38].

We searched for the sources of noise in the FS data. One possible source is that probes could be presented to the left or to the right eye, or to the more-sensitive or less-sensitive eye. These variables were not significant sources of noise (see Item S3, Table S1, Table S2, and Table S3).

**Figure 5:**
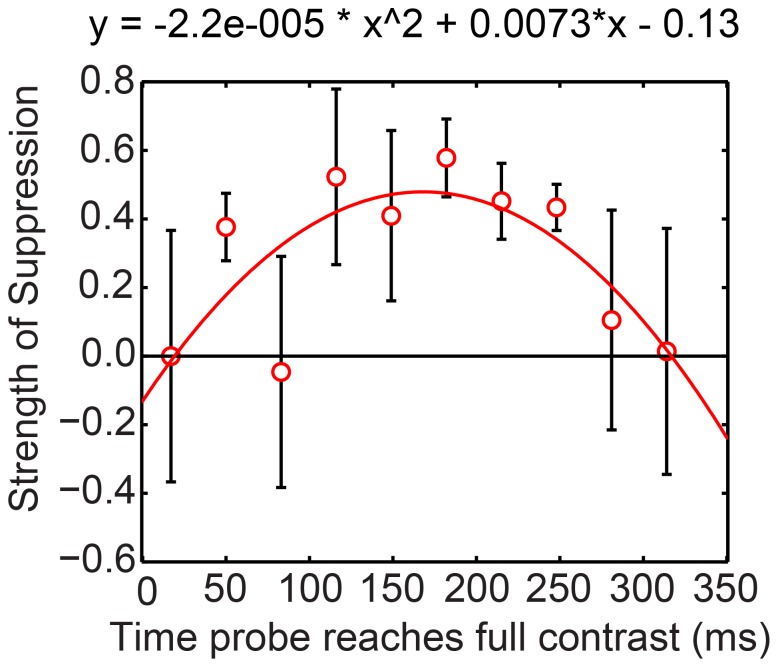
Temporal analysis of strength of suppression during swap interval of Experiment 2. Mean strength of suppression for probes as a function of when they reached maximum contrast after a swap during swap rivalry. Probes began about 50 ms earlier than this and persisted for about 50 ms after this. Probes reaching full contrast 283 ms or more after a swap were increasingly displayed during the early times after the next swap. The contrast increment was 0.27 (*n* = 5). The curve shows the significant quadratic function that explains some of the variability of the FS data.

Another possible source of noise could arise from Experiment 2’s fixed delay after a key press: rather than the probe’s appearing at a fixed time after the onset of a swap, as happened in Bhardwaj et al. [28], the probe could be presented at any time during a swap interval. To evaluate this, we grouped Experiment 2’s probe presentation times into 10 intervals every 33 ms after a swap and calculated sensitivities for each interval. Then we converted these sensitivities into strength of suppression. We have plotted mean strength of suppression against the midpoints of the intervals in Figure 5.

Figure 5 shows that, although noisy, strength of suppression shows a significant quadratic function against time during the swap interval, *t*(7) = –3.06, *p<*.05. That is:

- Suppression is weak for probes reaching full contrast in the first 75 ms after a swap.
- Suppression strengthens (except for one odd point for probes reaching full contrast 80 ms after a swap) to around 0.4, the usual value for conventional rivalry, until about 250 ms into the swap interval.
- Suppression becomes weaker again at longer times. For the last times we analysed, beginning at 280 ms after the swap, probes swapped eyes along with the test grating, so that an appreciable part of the time of these probes occurred in the next swap interval. Here we see the same pattern of results as immediately after the swap: very shallow, if any suppression.

We conducted the same analysis for three observers’ data at common contrast increments of 0.31, 0.35, and 0.39. With so few observers, these functions were noisier, but the same quadratic function was significant for a contrast increment of 0.35. Figure S8 shows these functions.

The results of this post-hoc analysis encouraged us to design Experiment 3 in which we manipulated the time probes were presented after a swap.

## Experiment 3

In Experiment 3, we introduced the probe either immediately after a swap (i.e., reaching full contrast 50 ms after the swap), in the middle of the swap interval, or 250 ms before the end of a swap interval. We chose the first time to replicate the time of probes used by Bhardwaj et al. [28]. We chose the last time to ensure that probes finished being shown 33 ms before the next swap, so as to minimize any involvement of that swap, through backwards masking, with detecting the probe. We chose the middle time to lie in between the other two times.

### Materials and Methods

The observers, apparatus, and stimuli were the same as in Experiment 2, except we tested only FO and FS rivalry. Each observer participated in at least thirteen sessions of data collection. A single session involved one run through the factorial design of the Experiment: two sorts of rivalry (FO vs FS) × two perceptual states (dominance vs. suppression) × three probe delays (0, 100, and 200 ms after the onset of the red stimulus to the left eye) for a total of 12 blocks of trials. Order of delays was randomised afresh in each session for each observer; the first rivalry condition for the first delay was chosen randomly for each observer then alternated; similarly, the first perceptual state to be tested for each observer was chosen randomly then alternated. We told observers only which orientation should be dominant before they pressed a key for each trial of a particular block.

In each block of trials, we measured a contrast increment threshold using a staircase procedure to find the 79% threshold level. The staircase decreased the contrast increment by 0.1 log units after every three consecutive correct responses, and increased it by the same amount after each wrong response. The staircase was preceded by four practice trials. The staircase was terminated after 10 reversals and the average of the last six reversals was taken to be the detection threshold. On average the staircase comprised 44±9 trials and took 3 minutes to complete.

The probe was a contrast increment applied to the red horizontal grating in the left eye. When the observer depressed a key, the program checked for the correct interval to show the probe. For example if it was a block in which the probe was to be presented at the start of the swap interval and the red horizontal grating had just swapped to the left eye then the probe was shown, otherwise the program waited for the next correct interval to show the probe. It was the same for the other conditions of probe timing; all involved a random delay between 13 ms (1 frame) and 666 ms (50 frames) after the key press, which is similar to Bhardwaj et al. [28] and to Experiment 1.

It is important to note that this random delay was the same for all three conditions of probe timing. The delay was governed by the relation between the participant’s key press and the time in the swap interval. For example, if the participant happened to press the key for a late-probe trial 14 ms before the time to show a late probe, that probe would be shown immediately, whereas if it was 12 ms before that time, the probe would have to wait until the next appropriate swap interval, 612 ms later.

The FO condition had a pseudo-swap code to mimic similar random delays in probe presentation. The order of the conditions was randomized within a session and the order of testing dominance and suppression was random for each observer, and then alternated over sessions.

### Results and Discussion

#### Strength of suppression

**Figure 6:**
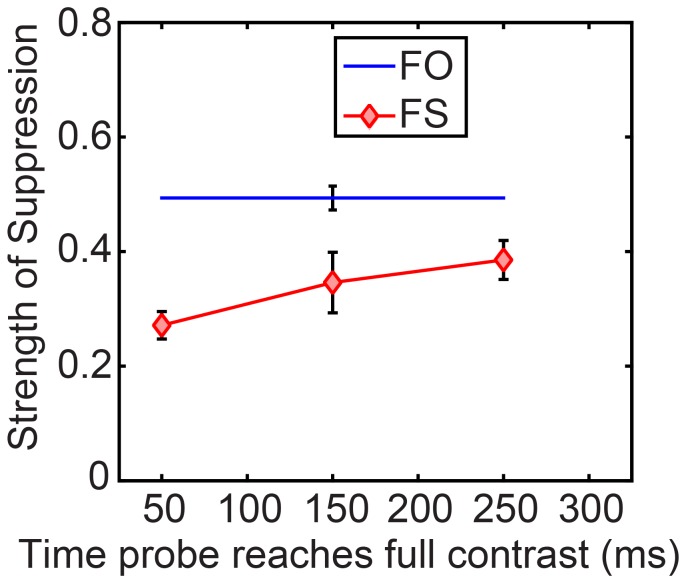
Strength of suppression for different rivalry conditions in Experiment 3 as a function of when they reached maximum contrast after a swap during swap rivalry (there was no swap for FO rivalry). Error bars show ±1 standard error of the mean. There is a significant strengthening of suppression with time in the swap interval.

We give details of the analysis of contrast-increment thresholds during dominance and during suppression (see Item S5, Figure S9 for mean data, and Figure S10’s left panel for individual data). From the contrast-increment thresholds we calculated strength of suppression using Equation 1, which can range from 0 (no suppression) to 1 (complete suppression). We plot mean strength of suppression for the two sorts of rivalry in Figure 6 (see Figure S10’s right panel for individual observer data). Note that we have shown only overall means for the FO conditions; because there was no swap, any differences among the three “times” arise only from sampling error. Figure 6 shows that suppression in FS rivalry increased in strength linearly with time after the swap, F(1,8) = 11.17, p = .01.

What we can conclude from Experiment 3 is that probe time in a swap interval has an effect on strength of FS rivalry suppression. When probes are presented immediately after the start of the swap interval, similar to Bhardwaj et al., then FS suppression is weaker than FO suppression. However, for probes presented later in the swap interval suppression during FS and FO is similar. What causes suppression to strengthen after a swap?

When conventional rivalry stimuli are switched on for the first time, one sees a combination of the two stimuli, no rivalry, for the initial 100 ms or so, after which perception resolves into one or the other image [39], [40]. We propose that this mainly involves the eyes: During the initial 100 ms, excitation of the two sets of neurons mediating the input from the eyes yields binocular fusion. But at the same time, inhibition between the two sets of neurons builds until by about 100 ms one set becomes completely inhibited, yielding suppression, and the other set becomes completely excited, yielding dominance. When swap-rivalry stimuli swap, we propose that the initial shallow suppression is from whatever mechanism mediates image rivalry and the subsequent deeper suppression is from adding the effects of eye rivalry.

An alternative explanation that could be imagined is that there is some form of masking following a swap that interferes with suppression. But this would require that the masking effect from a swap be different for a particular stimulus when it is suppressed from when it is dominant. Any general masking effect that is indifferent to perceptual state must be cancelled out when we calculate strength of suppression, because this is a ratio of the two perceptual states.

## General Discussion

From Experiment 1, we found that the results of Bhardwaj et al. [28] are reliable.

From Experiment 2 we found that:

- Poorer sensitivity during suppression than during dominance is not accompanied by any differences in response criteria for any condition, at least with our procedure of giving feedback on every trial. We conclude that highly trained observers can adopt the same response criteria for suppression and dominance, which means we do not have to worry about whether response criteria might affect responses in yes-no tasks. Our result is different to that of Caetta et al. [30], but whether this is from differences in the task (rivalry in our experiment, motion-induced blindness in that of Caetta et al.), differences in the location of the stimuli (central vs. peripheral), or differences in procedure (feedback after every trial vs. no feedback) remains to be learned.
- Strength of suppression is constant over the range of contrast increments.
- Strength of suppression increases over the first 150 ms or so after a swap during swap rivalry.

From Experiment 3, we confirmed that strength of suppression increases over the first 150 ms or so after a swap during swap rivalry. Bhardwaj et al. [28] reported suppression strength of about 0.4 for conventional rivalry and 0.2 for swap rivalry for probes beginning immediately after the swap. We found suppression strength of about 0.45 for conventional rivalry in Experiment 2 and in Experiment 3. We found strength of swap rivalry suppression to increase in all experiments from about 0.25 for probes reaching full contrast about 50 ms after the swap to about 0.4 for probes reaching full contrast about 150 ms after the swap.

Bhardwaj et al. [28] concluded that conventional rivalry suppression is mediated via both eye rivalry and by image rivalry. They also concluded that swap rivalry suppression is mediated weakly, if at all, at the early, eye-rivalry site and mainly at the later, image-rivalry site of the visual system. But the results of Experiments 2 and 3 reported here force some re-evaluation of this last conclusion.

Logothetis et al. [3] argued that swapping the rival stimuli between the eyes rules out any influence of eye rivalry. But we know that rivalry can develop within about 100 or so ms after onset of rival stimuli [39], [40], so strengthening eye rivalry throughout the swap interval is a possible explanation of our result that suppression strengthens within a swap interval. It may be that there is a baseline level of suppression from image rivalry, accomplished by higher-level neurons, that governs the suppression occurring immediately after a swap, and that these neurons, through feedback connections, entrain the lower-level neurons responsible for eye rivalry. If so, these modifications need to be made to models designed to explain the processing of conventional and swap rivalry [14], [41], [42].

There are different effects of timing on rivalry behaviour at longer time scales that need to be distinguished from our finding. For example, Bartels and Logothetis [43], using a flash suppression paradigm, evaluated the contribution of eye and image rivalry over time. They found that which eye is dominant determines perception 300 ms after the start of a rivalry suppression phase but that which image is dominant determines perception 3000 ms after the start. Even though the time range studied by Bartels and Logothetis [43] is much greater than that we studied in a single swap it does point to the fact that contribution from early (eye dependent) and late (eye independent) areas vary during the course of a single episode of suppression.

For another example, Alais et al. [44] showed that the temporal course of suppression during conventional rivalry decreases for an episode of suppression, over several seconds. The changes in suppression we are studying are much more fine grained: over one third of a second–the duration of a swap during swap rivalry–for which Alais et al. would not expect much change in strength of suppression from adaptation. Moreover, Alais et al. presented the rival stimuli continuously and delivered probes at random times, allowing the build-up of adaptation to continue over all of an episode of suppression, whereas we terminated the rival stimuli once the probe was presented, essentially resetting the state of adaptation before the next trial.

Bhardwaj et al. [28], from their finding of weak suppression during swap rivalry, pointed out that swap rivalry shares two suggestive similarities with monocular rivalry: First, neither requires the eye-of-origin information to be retained. Second, monocular-rivalry suppression is weak [45], around 0.1. However, this now needs to be reconsidered. Our current results suggest that eye-of-origin information is diminished but not abolished during swap rivalry. We now have evidence consistent with the notion that weak suppression just after the swap is because eye rivalry has not had a chance to develop, the swap between the eyes having disrupted it.

## Conclusion

We have shown that the decrease in sensitivity during rivalry suppression is not due to a change in response criteria. What we found to be critical is the timing of the probe after rival stimuli swap between the eyes: suppression is weak immediately after the swap and stronger 150 ms later. This suggests that image rivalry supports the initial weak suppression of swap rivalry and that eye rivalry augments it later in the swap interval. We propose that swap rivalry is not a pure form of image rivalry but involves eye-rivalry too.

## Supporting Information

Figure S1 **Thresholds and strengths of suppression for different rivalry conditions from Experiment 1.**
(EPS)

Figure S2 **Response criteria for different rivalry condition in Experiment 2.**
(EPS)

Figure S3 **Criteria for individual observers for different rivalry conditions in Experiment 2.**
(EPS)

Figure S4 **Sensitivity measures for individual observers for different rivalry conditions in Experiment 2.**
(EPS)

Figure S5 **Sensitivity measures for different contrasts and rivalry conditions in Experiment 2.**
(EPS)

Figure S6 **Individual observers’ sensitivities and strengths of suppression for the common contrast of 0.27 used in different rivalry conditions in Experiment 2.**
(EPS)

Figure S7 **Strength of suppression as a function of contrast for individual observers and different rivalry conditions in Experiment 2.**
(EPS)

Figure S8 **Mean strength of suppression for probes as a function of when they reached maximum contrast after a swap during swap rivalry in Experiment 2 for different values of contrast.**
(EPS)

Figure S9 **The mean threshold at the time probes reached peak contrast during a swap interval for all observers in Experiment 3 for flicker-only (FO; left panel) and flicker-and-swap (FS; right panel).**
(EPS)

Figure S10 **Mean contrast-increment thresholds and mean suppression strengths for individual observers in Experiment 3.**
(EPS)

Table S1 **A Four-factor ANOVA of Sensitivity (** ***d′*** **) from Experiment 2 for Eye of Presentation (** ***n*** **= 3).**
(DOCX)

Table S2 **A Three-factor ANOVA of Sensitivity (** ***d′*** **) from Experiment 2 for Common Contrast (.27) Value (** ***n*** **= 5) for Eye of Presentation.**
(DOCX)

Table S3 **A Three-factor ANOVA of Sensitivity (** ***d′*** **) from Experiment 2 for Common Contrast (.27) Value (** ***n*** **= 5) with More-sensitive Eye.**
(DOCX)

Item S1 **Analysis of sensitivity (** ***d′*** **) from Experiment 2.**
(DOCX)

Item S2 **Analysis of sensitivity from Experiment 2 at the common contrast increment of 0.27.**
(DOCX)

Item S3 **Analysis of eye of presentation from Experiment 2.**
(DOCX)

Item S4 **How we calculated the percentage of probes that need to be delivered to the incorrect state to halve the strength of suppression.**
(DOCX)

Item S5 **Analysis of log thresholds from Experiment 3.**
(DOCX)
