# Supplementary material for: Temporal Analysis of Image-Rivalry Suppression
Source: PLoS One. 2012 Sep 25;7(9):e45407. doi: 10.1371/journal.pone.0045407 (PMC3458036; doi:10.1371/journal.pone.0045407)
Supplement: Table S1 — A Four-factor ANOVA of Sensitivity ( d′ ) from Experiment 2 for Eye of Presentation ( n = 3). (DOCX) [file pone.0045407.s011.docx]

Table S1

*A Four-factor ANOVA of Sensitivity (d') from Experiment 2 for Eye of Presentation (*n *= 3)*

| Source | *df* | *SS* | *MS* | *F* |
| --- | --- | --- | --- | --- |
| Contrast | 6 | 133.825 | 22.304 | 43.547**** |
| Error (Contrast) | 12 | 6.146 | .512 |  |
| Rivaly condition | 2 | 122.026 | 61.013 | 56.824*** |
| Error (Rivaly condition) | 4 | 4.295 | 1.074 |  |
| Eye | 1 | 4.156 | 4.156 | 1.890 |
| Error (Eye) | 2 | 4.398 | 2.199 |  |
| State | 1 | 70.285 | 70.285 | 16.015* |
| Error (State) | 2 | 8.777 | 4.389 |  |
| Contrast * Rivaly condition | 12 | 50.894 | 4.241 | 17.681**** |
| Error (Contrast * Rivaly condition) | 24 | 5.757 | .240 |  |
| Contrast * Eye | 6 | .814 | .136 | .971 |
| Error (Contrast * Eye) | 12 | 1.676 | .140 |  |
| Contrast * State | 6 | 6.904 | 1.151 | 16.091**** |
| Error (Contrast * State) | 12 | .858 | .072 |  |
| Rivaly condition * Eye | 2 | 1.246 | .623 | 1.081 |
| Error (Rivaly condition * Eye) | 4 | 2.304 | .576 |  |
| Rivaly condition * State | 2 | 15.413 | 7.707 | 36.364** |
| Error (Rivaly condition * State) | 4 | .848 | .212 |  |
| Eye * State | 1 | .063 | .063 | .830 |
| Error (Eye * State) | 2 | .153 | .076 |  |
| Contrast * Rivaly condition * Eye | 12 | 2.661 | .222 | .931 |
| Error (Contrast * Rivaly condition * Eye) | 24 | 5.717 | .238 |  |
| Contrast * Rivaly condition *State | 12 | 7.225 | .602 | 2.741* |
| Error (Contrast * Rivaly condition *State) | 24 | 5.272 | .220 |  |
| Contrast * Eye * State | 6 | .451 | .075 | .350 |
| Error (Contrast * Eye * State) | 12 | 2.576 | .215 |  |
| Rivaly condition * Eye * State | 2 | .027 | .014 | .049 |
| Error (Rivaly condition * Eye * State) | 4 | 1.117 | .279 |  |
| Contrast * Rivaly condition * Eye * State | 12 | 3.257 | .271 | 1.278 |
| Error (Contrast * Rivaly condition * Eye * State) | 24 | 5.098 | .212 |  |

Note: ⃰ *p* < .05, ⃰ ⃰ *p* < .01, ⃰ ⃰ ⃰ *p* <.001, ⃰ ⃰ ⃰ ⃰ *p* < .0001
