# Supplementary material for: Temporal Analysis of Image-Rivalry Suppression
Source: PLoS One. 2012 Sep 25;7(9):e45407. doi: 10.1371/journal.pone.0045407 (PMC3458036; doi:10.1371/journal.pone.0045407)
Supplement: Table S2 — A Three-factor ANOVA of Sensitivity ( d′ ) from Experiment 2 for Common Contrast (.27) Value ( n = 5) for Eye of Presentation. (DOCX) [file pone.0045407.s012.docx]

Table S2

*A Three-factor ANOVA of Sensitivity (d') from Experiment 2 for Common Contrast (.27) Value (*n *= 5) for Eye of Presentation*

| Source | *df* | *SS* | *MS* | *F* |
| --- | --- | --- | --- | --- |
| Rivaly condition | 2 | 106.233 | 96.792 | 68.042*** |
| Error (Rivaly condition) | 8 | 6.245 | 0.781 |  |
| Eye | 1 | .217 | .217 | .404 |
| Error (Eye) | 4 | 2.148 | .537 |  |
| State | 1 | 20.498 | 20.498 | 25.634** |
| Error (State) | 4 | 3.199 | .800 |  |
| Rivaly condition * Eye | 2 | .027 | .013 | .165 |
| Error (Rivaly condition * Eye) | 8 | .645 | .081 |  |
| Rivaly condition * State | 2 | 7.718 | 3.859 | 14.831** |
| Error (Rivaly condition * State) | 8 | 2.082 | .260 |  |
| Eye * State | 1 | .008 | .008 | .119 |
| Error (Eye * State) | 4 | .267 | .067 |  |
| Rivaly condition * Eye * State | 2 | .018 | .009 | .156 |
| Error (Rivaly condition * Eye * State) | 8 | .462 | .058 |  |

Note: ⃰ *p* < .05, ⃰ ⃰ *p* < .01, ⃰ ⃰ ⃰ *p* <.001, ⃰ ⃰ ⃰ ⃰ *p* < .0001
