# Supplementary material for: Temporal Analysis of Image-Rivalry Suppression
Source: PLoS One. 2012 Sep 25;7(9):e45407. doi: 10.1371/journal.pone.0045407 (PMC3458036; doi:10.1371/journal.pone.0045407)
Supplement: Table S3 — A Three-factor ANOVA of Sensitivity ( d′ ) from Experiment 2 for Common Contrast (.27) Value ( n = 5) with More-sensitive Eye. (DOCX) [file pone.0045407.s013.docx]

Table S3

*A Three-factor ANOVA of Sensitivity (d') from Experiment 2 for Common Contrast (.27) Value (*n *= 5) with More-sensitive Eye*

| Source | *df* | *SS* | *MS* | *F* |
| --- | --- | --- | --- | --- |
| Rivaly condition | 2 | 106.233 | 53.116 | 68.042**** |
| Error (Rivaly condition) | 8 | 6.245 | .781 |  |
| More-sensitive eye | 1 | 1.224 | 1.224 | 4.291† |
| Error (More-sensitive eye) | 4 | 1.141 | .285 |  |
| State | 1 | 20.498 | 20.498 | 25.634** |
| Error (State) | 4 | 3.199 | .800 |  |
| Rivaly condition * More-sensitive eye | 2 | .123 | .062 | .897 |
| Error (Rivaly condition * More-sensitive eye) | 8 | .549 | .069 |  |
| Rivaly condition * State | 2 | 7.718 | 3.859 | 14.831** |
| Error (Rivaly condition * State) | 8 | 2.082 | .260 |  |
| More-sensitive eye * State | 1 | .001 | .001 | .009 |
| Error (More-sensitive eye * State) | 4 | .275 | .069 |  |
| Rivaly condition * More-sensitive eye * State | 2 | .016 | .008 | .141 |
| Error (Condition * More-sensitive eye * State) | 8 | .464 | .058 |  |

Note: † *p* = .10, ⃰ *p* < .05, ⃰ ⃰ *p* < .01, ⃰ ⃰ ⃰ *p* <.001, ⃰ ⃰ ⃰ ⃰ *p* < .0001
