# Supplementary material for: Temporal Analysis of Image-Rivalry Suppression
Source: PLoS One. 2012 Sep 25;7(9):e45407. doi: 10.1371/journal.pone.0045407 (PMC3458036; doi:10.1371/journal.pone.0045407)
Supplement: Item S1 — Analysis of sensitivity ( d′ ) from Experiment 2. (DOCX) [file pone.0045407.s014.docx]

*Item S1 Analysis of sensitivity (d') from Experiment 2*

To analyse the sensitivity results from Experiment 2, we pooled the data from the 10 sessions to calculate the *d'* for each observer in all the conditions so that each value for each observer was based on 500 trials. We analysed these using a three-way within-subject ANOVA with condition (static / FO / FS), contrast (with seven levels of increments), and state (dominance / suppression) as factors. We give individual observer data in Figure S4 and mean data in Figure S5’s right panel.

The ANOVA yielded the following main effects and interactions:

- Sensitivity grows linearly with probe contrast, *F*(1, 2) =159.52, p < .0001. This is not a particularly surprising finding. It means that sensitivity improved with greater signal strength of the probe.
- Sensitivity is higher in the static condition than in the FO and FS conditions, F(1, 2) = 245.02, p < .0001. This probably occurs because the 18 Hz flicker acts as a temporal mask for the probe [Supporting reference 1].
- The improvement in sensitivity with contrast is greater in static than in FO and FS conditions, F(12, 24) = 25.22, p < .0001. This also probably arises because of the temporal masking by the flicker.
- Critically, the increase in sensitivity with increasing contrast is higher during dominance than during suppression, F(6, 12) = 7.75, p < .001. This confirms and extends the classic finding that probes are easier to see during dominance than during suppression [22, 24, 25, 26, 28]. This difference is greater in static than in FO and FS conditions, F(2, 4) = 15.19, p < .01, as is the rate of increase with contrast. Although the difference between dominance and suppression sensitivities is greater for FO than for FS (consistent with 29), it is not significant, F(1, 2) = 0.088, p > .05.
- There was a significant three-way interaction, F(12, 24) = 6.44, p < 0.0001, indicating that the earlier-mentioned differences are more for static rivalry than for the other two sorts of rivalry (Figure S5, left panel).

Supporting Reference

1. Cass J, Alais D (2006). Evidence for two interacting temporal channels in human visual processing. Vision Research 46: 2859-2868.
