# Supplementary material for: Temporal Analysis of Image-Rivalry Suppression
Source: PLoS One. 2012 Sep 25;7(9):e45407. doi: 10.1371/journal.pone.0045407 (PMC3458036; doi:10.1371/journal.pone.0045407)
Supplement: Item S2 — Analysis of sensitivity from Experiment 2 at the common contrast increment of 0.27. (DOCX) [file pone.0045407.s015.docx]

Item S2 *Analysis of sensitivity from Experiment 2 at the common contrast increment of 0.27*

A contrast increment of 0.27 was common for all the conditions and observers. To confer greater statistical power we ran two new observers (AP and FR) at the same contrast increment and combined all observers’ results into a two-way within-subject ANOVA with condition (static / FO / FS) and state (dominance / suppression) as factors (see Figure S6’s top panel for individual observer data and Figure S5’s right panel for mean data). This yielded two significant main effects and a significant two-way interaction:

- The main effect of condition was significant, *F*(2, 8) = 57.07, *p* < 0.0001: sensitivity is higher during static rivalry than during the other two conditions F(1,4) = 67.953, *p* < 0.001.
- The main effect of state was also significant, *F*(1, 4) = 25.27, *p* < .01, indicating higher sensitivity during dominance than during suppression.
- The significant two-way interaction, *F*(2, 8) = 19.09, *p* < 0.001, shows that the higher sensitivity during dominance than during suppression, was greater for static conditions than for the other two conditions (Figure S5, left panel).
