# Supplementary material for: Temporal Analysis of Image-Rivalry Suppression
Source: PLoS One. 2012 Sep 25;7(9):e45407. doi: 10.1371/journal.pone.0045407 (PMC3458036; doi:10.1371/journal.pone.0045407)
Supplement: Item S3 — Analysis of eye of presentation from Experiment 2. (DOCX) [file pone.0045407.s016.docx]

*Item S3 Analysis of eye of presentation from Experiment 2*

Because we presented the contrast increment with a fixed delay after a key-press, sometimes the to-be-probed stimulus was viewed by the left eye, sometimes by the right, and sometimes it swapped between the eyes. To test the effect of eye, we grouped all data according to which eye received the probe (left or right eye). We conducted a four-way within-subject ANOVA on sensitivity with condition (static / FO / FS), contrast (with seven levels of increments), state (dominance / suppression) and eye of presentation (LE / RE) as factors. Neither the main effect of eye nor any of its interactions was significant indicating that sensitivity was not affected by which eye received the probe (Table S1). We conducted a similar analysis for the common contrast increment of 0.27. This analysis showed no effect of eye (Table S2). We also conducted the same analysis with the eye factor replaced by one with levels of more-sensitive vs less-sensitive eye. This factor did not interact with any other factor (Table S3).

Because the more sensitive eye of any observer could differ, we plotted individual observers’ data to find which eye had better sensitivity. The left eye had better sensitivity for observers MJB, LC, and RB; the right eye had better sensitivity for observers AP and FR. We then repeated the analysis replacing the Eye factor by More-sensitive eye (see Table S3).
