# Supplementary material for: Temporal Analysis of Image-Rivalry Suppression
Source: PLoS One. 2012 Sep 25;7(9):e45407. doi: 10.1371/journal.pone.0045407 (PMC3458036; doi:10.1371/journal.pone.0045407)
Supplement: Item S4 — How we calculated the percentage of probes that need to be delivered to the incorrect state to halve the strength of suppression. (DOCX) [file pone.0045407.s017.docx]

*Item S4 How we calculated the percentage of probes that need to be delivered to the incorrect state to halve the strength of suppression*

We used simulation to derive the percentage of probes that need to be delivered to the incorrect state to halve the strength of suppression. We generated 100 experiments that yielded sensitivities for suppression and dominance of 3 and 5 respectively. Each experiment gives strength of suppression of 0.4 (Equation 1.1). Then we reversed these sensitivities (i.e., 5 for “suppression” and 3 for “dominance”) for various numbers of experiments until the average suppression strength over all experiments was 0.2. Nineteen such reversed experiments were necessary. That is, probes would have to be delivered to the wrong state on 19% of trials to halve strength of suppression.

In fact, 19% of trials in which probes are delivered to the wrong state is the minimum percentage because of the incidence of mixed states. Our observation is that these are much more frequent during swap rivalry than during conventional rivalry. We assumed that when a probe fell into a mixed state, sensitivity would be equal to 5 for “suppression” and “dominance”. We also assumed that there were as many examples of probes being delivered to mixed states as to incorrect states. With these assumptions, 27% of probes would have to be delivered to the wrong state to halve strength of suppression, which is even less likely.
