# Supplementary material for: Temporal Analysis of Image-Rivalry Suppression
Source: PLoS One. 2012 Sep 25;7(9):e45407. doi: 10.1371/journal.pone.0045407 (PMC3458036; doi:10.1371/journal.pone.0045407)
Supplement: Item S5 — Analysis of log thresholds from Experiment 3. (DOCX) [file pone.0045407.s018.docx]

**Experiment 3**

*Item S5 Analysis of log thresholds from Experiment 3*

In our initial statistical analyses of contrast-increment thresholds, we found that their variances were correlated with their values, violating the assumption of homogeneity of variance made by ANOVA. To restore homogeneity of variance, we log-transformed contrast thresholds.

We conducted a within-subject ANOVA on log thresholds with probe time (start, 50 ms / middle, 150 ms / end, 250 ms), condition (FO / FS), and state (dominance / suppression) as factors. We give mean data in Figure S9 and individual observer data in Figure S10’s left panel. We found the following significant main effects and interactions:

- Thresholds were higher during FS conditions than during FO conditions, *F*(1, 4) = 17.17, *p* < .01, which replicates the patterns of results found by Bhardwaj et al. [28].
- Thresholds during suppression were higher than during dominance, *F*(1, 4) = 50.31, *p* < .01 , again replicating the results of Bhardwaj et al. [28] and numerous other authors [14, 22, 24, 25].
- The difference between dominance and suppression thresholds was greater in FO conditions than in FS conditions, *F*(1, 4) = 7.82, *p* = .05. The average threshold contrast for FO conditions was 0.1 during dominance and 0.3 during suppression. The average threshold contrast for FS conditions was 0.2 during dominance and 0.3 during suppression. Critically, this difference was greater in FO conditions than FS conditions for probes presented at the start of the swap interval (i.e., probes reaching full contrast 50 ms after the swap) than for probes presented either at the middle of the swap interval (150 ms) or near the end of the swap interval (250 ms).
- There was a significant three-way interaction, *F*(2, 4) = 20.29, *p* < .001 (see Figure S9) indicating that thresholds for suppression in the FS condition grow with time in the swap interval, whereas thresholds for dominance stay the same, and that the difference between the thresholds for suppression and dominance in the FO condition stays the same. This interaction yields the significant effect on strength of suppression shown in Figure 3 of the main text.
